# Differential microRNA Expression and Regulation in the Rat Model of Post-Infarction Heart Failure

**DOI:** 10.1371/journal.pone.0160920

**Published:** 2016-08-09

**Authors:** Xueyan Liu, Heyu Meng, Chao Jiang, Sibao Yang, Fengwen Cui, Ping Yang

**Affiliations:** 1 Department of Internal Medicine and Cardiology, China–Japan Union Hospital of Jilin University, Changchun, China; 2 Clinical Medicine, Yanbian University, Yanji, China; 3 Department of Hepatobiliary Pancreatic Surgery, First Hospital of Jilin University, Changchun, China; The University of Tennessee Health Science Center, UNITED STATES

## Abstract

**Background:**

Heart failure is a complex end stage of various cardiovascular diseases with a poor prognosis, and the mechanisms for development and progression of heart failure have always been a hot point. However, the molecular mechanisms underlying the post transcriptional regulation of heart failure have not been fully elucidated. Current data suggest that microRNAs (miRNAs) are involved in the pathogenesis of heart failure and could serve as a new biomarker, but the precise regulatory mechanisms are still unclear.

**Methods:**

The differential miRNA profile in a rat model of post-infarction heart failure was determined using high throughout sequencing and analyzed through bioinformatics approaches. The results were validated using qRT-PCR for 8 selected miRNAs. Then the expression patterns of 4 miRNAs were analyzed in different periods after myocardial infarction. Finally, gain- and loss-of-function experiments of rno-miR-122-5p and rno-miR-184 were analyzed in H_2_O_2_ treated H9c2 cells.

**Results:**

In the heart failure sample, 78 miRNAs were significantly upregulated and 28 were downregulated compared to the controls. GO and KEGG pathway analysis further indicated the likely roles of these miRNAs in heart failure. Time-course analysis revealed different expression patterns of 4 miRNAs: rno-miR-122-5p, rno-miR-199a-5p, rno-miR-184 and rno-miR-208a-3p. Additionally, rno-miR-122-5p and rno-miR-184 were proved to promote apoptosis in vitro.

**Conclusions:**

Differential profile and expression patterns of miRNAs in the rats model of post-infarction heart failure were found, and the pro-apoptotic roles of rno-miR-122-5p and rno-miR-184 were revealed. These findings may provide a novel way that may assist in heart failure diagnosis and treatment.

## Introduction

Heart failure(HF) is one of the common end stages of cardiovascular diseases with a poor prognosis highlighted by a 5-year mortality of nearly 70%[1]. HF is the response to injury caused by significant ventricular remodeling, and is characterized by cardiac dysfunction, cardiomyocyte apoptosis, upregulation of fetal gene expression, impaired myocardial vascularization, unfavorable changes in extracellular matrix composition and fibrosis[2–4]. Although the mechanisms for development and progression of HF have been extensively studied, the molecular mechanisms underlying the post transcriptional regulation of HF have not been fully elucidated.

MicroRNAs (miRNAs) are small, non-coding regulatory RNA molecules that either promote degradation or suppress the translation of their target mRNAs with full or partial complementary sequences[5]. Thus far, 2588 mature unique miRNAs (miRbase release 21, June 2014) have been identified in human, 1915 in mice, and 765 in rats. In human, miRNAs target approximately 60% of protein coding genes[6]. Most miRNAs are evolutionarily conserved in vertebrates and play crucial roles in a variety of cellular and physiological activities, such as cell growth, proliferation, apoptosis, hypertrophy and excretion. However, precise regulatory mechanisms of most miRNAs remain unclear.

Accumulating evidences suggest that miRNAs may play an important role in the pathogenesis of heart failure through regulating the expression levels of related genes in cardiac remodeling. Cardiomyocyte-specific deletion of *decr8* in the mice, a gene required for miRNA biogenesis, revealed a progression of left ventricular dysfunction[7]. In the first miRNA deletion animal model in 2007, miR-208, a cardiac-specific miRNA, was found to be required for cardiomyocyte hypertrophy and fibrosis[8]. Furthermore, therapeutic silencing of miR-208a via subcutaneous delivery of antimiR-208a prevents pathological cardiac remodeling, functional deterioration, and lethality during heart disease, which indicated the potential therapeutic roles of modulating cardiac miRNAs during heart failure[9].

Recently, several expression profile studies using cloning or microarray approaches have identified certain miRNAs differentially expressed in HF caused by dilated cardiomyopathy[10, 11] in human hearts, but the differential miRNA profile of infarction induced heart failure has not been illuminated. For the present study, we employed Solexa deep sequencing technology to extend the repertoire of heart miRNAs in rats and compared the miRNA profile changes in post-infarction heart failure. Furthermore, the dynamic changes of miRNA expression patterns in different periods post-MI were investigated in vivo and the effects of two dysregulated miRNAs on cardiomyocyte apoptosis were explored in vitro. Consequently the possible regulatory mechanisms of miRNA in HF were discussed.

## Materials and Methods

### Establishment and evaluation of rat model of HF

Female wistar rats (n = 50, weighing 210±10g) were obtained from the Center for Laboratory Animals, Medical College, Jilin University, China. They were housed 2–3 per cage in a controlled environment (21°C±1°C, 45%-50% relative humidity, fixed 12-hour light/dark cycle). As previously described[12], after three-day acclimation, animals were fixed on an operating table after anesthetized by diethyl ether. An incision of the skin and intercostal muscles was made between the third and fourth ribs. A thoracotomy was performed and the pericardium was opened, which left the heart adequately exposed. For the operation group (n = 35), left anterior descending (LAD) coronary arteries were ligated with silk sutures. Then, the heart was returned to its normal position, the muscles and skins were sutured immediately. The sham-operated animals (the control group, n = 15) underwent the same procedure except that the silk suture was placed around the left coronary artery without being tied. After the surgery, all animals were injected with penicillin for three days to prevent infection. Eight rats with induced myocardial infarction died during or shortly after the operation and no rats in the sham group died. All rats were fed with standard diet and tap water. For both the control and operation groups, the rats were randomly selected to execute in the 4, 8 and 10 week after surgery (MI-4, MI-8, HF and Control). All animals received humane care and the experimental procedures were approved by the Animal Ethics Committee of Jilin University.

Echocardiography (Phillips HD7) was performed before execution. Under anesthesia by diethyl ether, rats were fixed on their backs with fur shaved and skin cleaned. Using a high-frequency linear-array transducer, the structural and functional parameters of the heart were examined and recorded, including heart rate (HR), interventricular septal thickness in diastole (IVSd), interventricular septal thickness in systole (IVSs), left ventricular internal dimension in diastole (LVIDd), left ventricular internal dimension in systole (LVIDs), left ventricular posterior wall thickness in diastole (LVPWd), left ventricular posterior wall thickness in systole (LVPWs), ejection fraction (EF) and fractional shortening (FS). To make the EF difference more intuitive, we calculate ratio of HF group to control group in EF value. EF ratio = EF_HF_/EF_control_×100%

Rats were executed death through over dosage of inhalation anesthetics. Hematoxylin and eosin (HE) staining and masson’s trichrome staining were performed to evaluate morphological changes and myocardial fibrosis in the left ventricular (LV) tissue of control, MI-4, MI-8 and HF group respectively. The detailed procedures are as the former reports[13]. In order to evaluate the apoptosis in vivo, immunohistochemical staining for caspase 3 was performed according to Yang et al[14]. Beside, caspase 3 activity colorimetric assay kit (BestBio, Beijing, China) was used according to the kit instructions.

Blood samples were collected in EDTA tubes, which were then places on ice and centrifuge within 30 min at 4°C. Enzyme linked immunosorbent assay (ELISA) for rat nt-proBNP was performed.

### Total RNA isolation and small RNA library sequencing

The non-infarcted left ventricular tissues were dissected and frozen in liquid nitrogen immediately and stored at -80°C.As described by Lian et al[15], total RNA was extracted using Trizol reagent (Invitrogen, Carlsbad, CA, USA) according to the manufacturer’s protocol, and the RNA concentration and purity were determined by the Agilent Technologies 2100 Bioanalyzer.

For small RNA library construction, total RNA isolated from the left ventricular tissues of the HF group and the Control group in the 10^th^ week after operation were pooled and prepared according to the Solexa EAS Small RNA Sample Prep Protocol. In brief, Solexa sequencing was performed as follows: For each library, approximately 10μg total RNA was size-fractionated on a 15% Tris /borate /EDTA urea denaturing PAGE gel to enrich for molecules in the range of 18–30 nucleotides in length and ligated with proprietary adaptors to the 5’ and 3’ termini of the RNA with T4 RNA ligase (Ambion, Austin, TX, USA). The adaptor-ligated small RNA was then converted to single-stranded cDNA using Superscript II reverse transcriptase (Invitrogen, Carlsbad, CA, USA). The resulting cDNA was amplified with 15PCR cycles using Illumina’s small RNA primer sets. The purified PCR products were quantified on an Agilent Technologies 2100 Bioanalyzer and diluted to 10 nM for sequencing on the Genome Analyzer GA-I (Illumina, San Diego, CA, USA) at the Beijing Genomics Institute (BGI, Shenzhen, China) according to the manufacturer’s protocol.

### Analysis of sequencing data

Raw sequence reads were processed into clean reads using the BGI small RNA reads pipeline as previously reported[16]. First, all low-quality reads were removed from the raw sequence reads. Then, the 3’ adaptor sequences were trimmed and the 5’ adaptor contaminations, sequences containing the polyA tail as well as those smaller than 18 nucleotides, were also discarded. The remaining 18–30 nucleotide high-quality identical sequences were counted, and the unique sequences with their associated read counts were mapped to the Norway rat genome assembly with no mismatch by the soap program[17]. All clean reads were annotated as one of the known classes of small RNA based on their overlap with publicly available genome annotations. To identify sequence tags originating from repeats, rRNA, tRNA, small nuclear RNA (snRNA) and small nucleolar RNA (snoRNA), we used the Rfam and NCBI GenBank databases (http://www.ncbi.nlm.nih.gov/). To identify known Rattus norvegicusmiRNAs, unique sequences were aligned with precursor miRNA sequences from miRBase 14.0 [18]. To avoid repeat annotation, these unique sequences were traversed in the order rRNA etc. (rRNA, tRNA, snRNA, snoRNA) >known rat miRNAs> repeat-associated small RNAs. To identify conserved miRNA homologs in rats, all clean sequences were additionally blastn (with a maximum of two mismatches) searched against the currently known human, mouse and rat mature miRNAs deposited in the miRBase(release 14.0)[18]. All unannotated small RNA sequences were also searched against known piRNAs retrieved from the RNAdb (http://jsm-research.imb.uq.edu.au/rnadb/default.aspx) with blastn, and only perfectly matched sequences were considered as candidate piRNAs. Potentially novel miRNAs were identified by folding the flanking genome sequence of unique small RNAs using mireap (https://sourceforge.net/projects/mireap/). To compare the differential expression of miRNAs between the two libraries, the Bayesian method developed for the analysis of digital gene expression profiles was used[19]. For more reasonable comparisons of both libraries, the count of the most abundant isomiR of each library was normalized against total counts of all known miRNAs detected in this library. miRNA target prediction and gene functional annotation was performed using the miRGen database [20] (http://www.diana.pcbi.upenn.edu/cgi-bin/miRGen/v3/Targets.cgi) and the DAVID gene annotation tool (http://david.abcc.ncifcrf.gov/), respectively.

### Stem-loop quantitative RT-PCR

A real-time quantification assay for miRNA was conducted as previously described[21]. Briefly, the assay was performed using stem-loop RT followed by quantitative PCR. First, 1 μg total RNA was reverse-transcribed to cDNA using ReverTra Ace reverse transcriptase (Toyobo Co.,Osaka, Japan) and miRNA-specific stem-loop RT primer. The mix was incubated at 37°C for 15 min, 85°C for 5 min and then held at 4°C using an Applied Biosystems 9700 Thermocycler. Then, quantitative PCR was performed on the Agilent TechnologiesMx3000P /Mx3005P Real-Time PCR Detection System by using a standard SYBR Green Real-time PCR Master Mix(Toyobo: QPK-201). In each reaction, 25 μL reaction mixtures containing 1 μL cDNA (1: 10 dilution) were prepared and incubated at 95°C for 5 min, followed by 40 cycles of 95°C for 15 s and 60°C for 45 s in a 96-well optical plate. The melting curve analysis and agarose gel electrophoresis were used to confirm the specific PCR products. All reactions were run in triplicate and porcine U6 snRNA was used as an endogenous reference. To calculate the expression level differences of miRNAs between samples examined, the △△Ct method was used [22]. Three independent samples were analyzed for each rat.

### Regulation of miRNAs in vitro

During the pathological progress of myocardial infarction induced heart failure, oxidative stress contributes to ventricular remodeling. H_2_O_2_ was used to set the HF model in vitro H9c2 cells. H9c2 embryonic rat myocardium-derived cells, a cell line that conserves the biological features of myocytes to study myocardial cell ischemia, hypertrophy and apoptosis[23], were kindly provided by the central laboratory of China-Japan union hospital, Jilin University. The cells were cultured in DMEM (Dulbecco’s Modified Eagle) medium supplemented with 10% fetal bovine serum at 37°C under 5% CO_2_.Cells were plated at a concentration of 3–8×10^5^/well in 6-well plates and cultured 24 hours to reach 70–90% confluence. Then they were treated with 200 nM H_2_O_2_ for 24 hours respectively. To examine the effects of rno-miR-122-5p and rno-miR-184, cells were transfected with rno-miR mimics, rno-miR inhibitors, or scrambled controls (Guangzhou Ruibo biology Science & Technology Co,; Ltd; China).Thirty hours after transfection, the cells were tested for the transfection efficiency and harvested.

Flow cytometry analysis was performed to measure H9c2cell apoptosis with Annexin-V-FLUOS Staining. Cells were analyzed using 488 nm excitation, a 515 nm band pass filter for fluorescein detection and a filter >600 nm for PI detection. Each treatment was performed three times. The results were expressed as means ± SD of the separate samples.

### Statistical Analysis

Data were analyzed with the SPSS16.0 version. All data were expressed as means ± SD. Comparisons were made between different groups using ANOVA followed by the Dunnett post hoc test for differences. The data for percent changes were analyzed using the Kruskal-Wallis *H-test*. A value of *P*<0.05 was considered statistically significant. All experiments conformed to the Chinese Academy of Medical Sciences ethics code of practice.

## Results

### General condition of HF and control rats model

**Fig 1:**
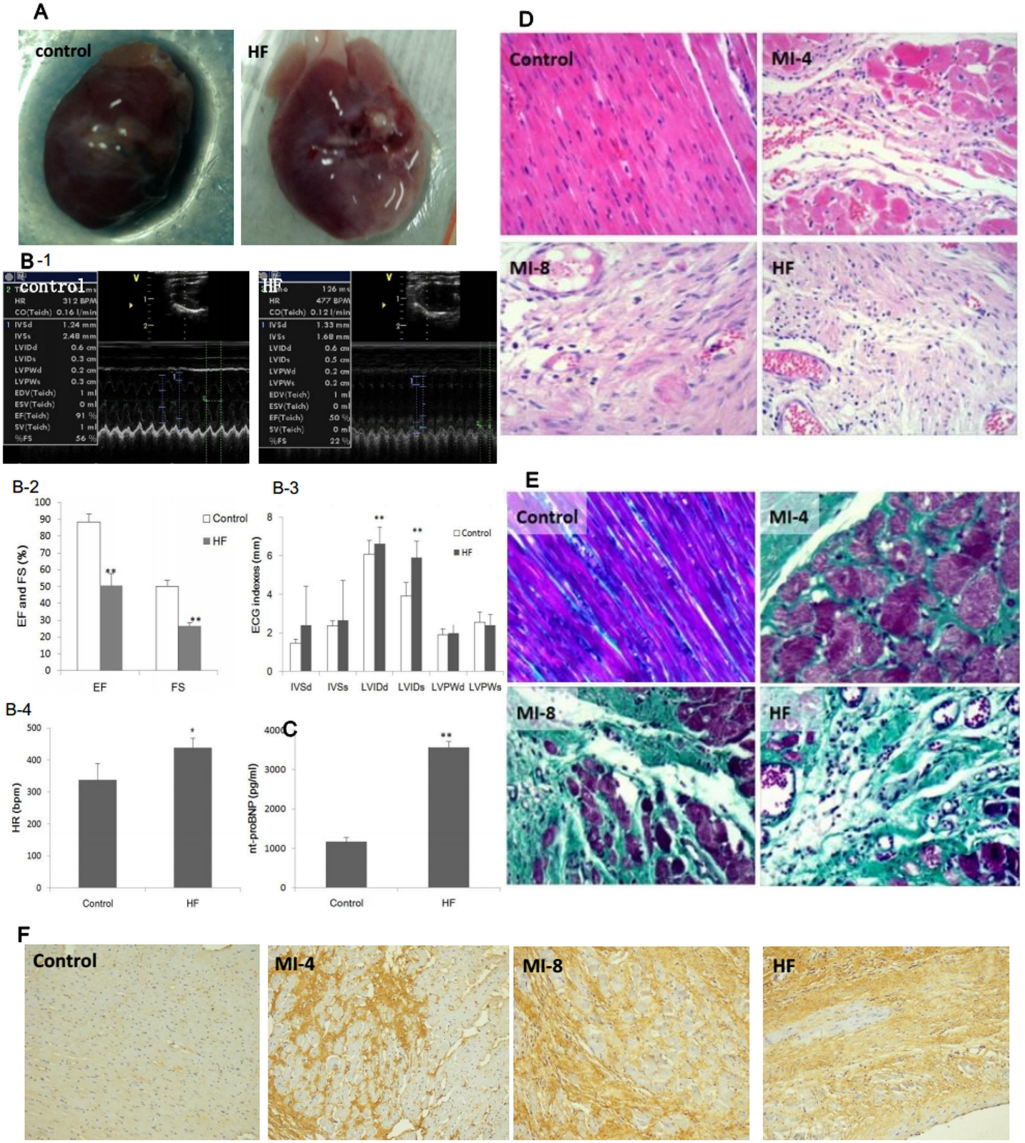
Evaluation of the heart functions of HF and control rats. **(A)** Representative images of HF and control hearts. **(B)** Echocardiographic index, including EF, FS, IVSd, IVSs, LVIDd, LVIDs, LVPWd, LVPWs and HR. EF, ejection fraction; FS, fractional shortening; IVSd, interventricular septal thickness in diastole; IVSs, interventricular septal thickness in systole; LVIDd, left ventricular internal dimension in diastole; LVIDs, left ventricular internal dimension in systole; LVPWd, left ventricular posterior wall thickness in diastole; LVPWs, left ventricular posterior wall thickness in systole; HR, heart rate.**(C)** The levels of plasma nt-proBNP in HF and control rats. **(D)** Hematoxylin and eosin (HE) staining of left ventricular (LV) tissue showed pathological and morphological changes in control, MI-4, MI-8 and HF group (magnification, ×200). **(E)** Masson’s trichrome staining of LV tissure showed progressively cardiac interstitial and perivascular fibrosis in MI-4, MI-8 and HF group compared to control group (magnification, ×200). **(F)** Immunohistochemical staining of Caspase 3 in LV tissue showed apoptosis in MI-4, MI-8 and HF group (magnification, ×200). Data were presented as means ± SD. **P<0.01 and *P<0.05.

A rat model of HF after myocardial infarction(MI) was established and the morphological changes between the HF group and control group were examined. Ten weeks after left anterior descending(LAD) ligation, the hearts in the HF group showed abnormal, ventricular chamber with large, fibrotic scar in the anterior wall of left ventricle (Fig 1A). Meanwhile, the echocardiographic assessment showed decreased EF, FS, and CO as well as increased HR and LVIDs (Fig 1B). Serum nt-proBNP level, a specific marker of HF, was significantly promoted in the HF group (P<0.01)(Fig 1C). The HE staining results showed that the myocardial cells from the control group were neatly arranged, and cross-striations appeared clearly. While in the MI-4 and the MI-8 group, the morphology and cell arrangement were more and more irregular, and in the HF group, besides the change mentioned above, considerable fibrous tissue proliferation appeared (Fig 1D). It can be observed from the Masson’s trichrome staining results that there was the least area of green-stained collagen fiber tissue between the myocardial cells in the control group; whereas there were progressively more green-stained collagen fibers in theMI-4, MI-8 and HF groups (Fig 1E). EF ratio was 54.9%, which indicated the ejection function of HF rats was 54.9% of the control rats. Caspase 3 is a key factor in the apoptosis pathway, and the expression level is positively correlated with apoptosis. We evaluated caspase 3 expressions in vivo through immunocytochemistry which showed that caspase 3 expression was increased in the MI-4, MI-8 and HF group compared with the control group (Fig 1F). Caspase 3 activity in HF group was 151.5% of the control group (p<0.05). These results suggest that post-infarction heart failure was accompanied by progressive myocardial fibrosis and apoptosis.

### Small RNA sequencing, annotation and analysis

**Fig 2:**
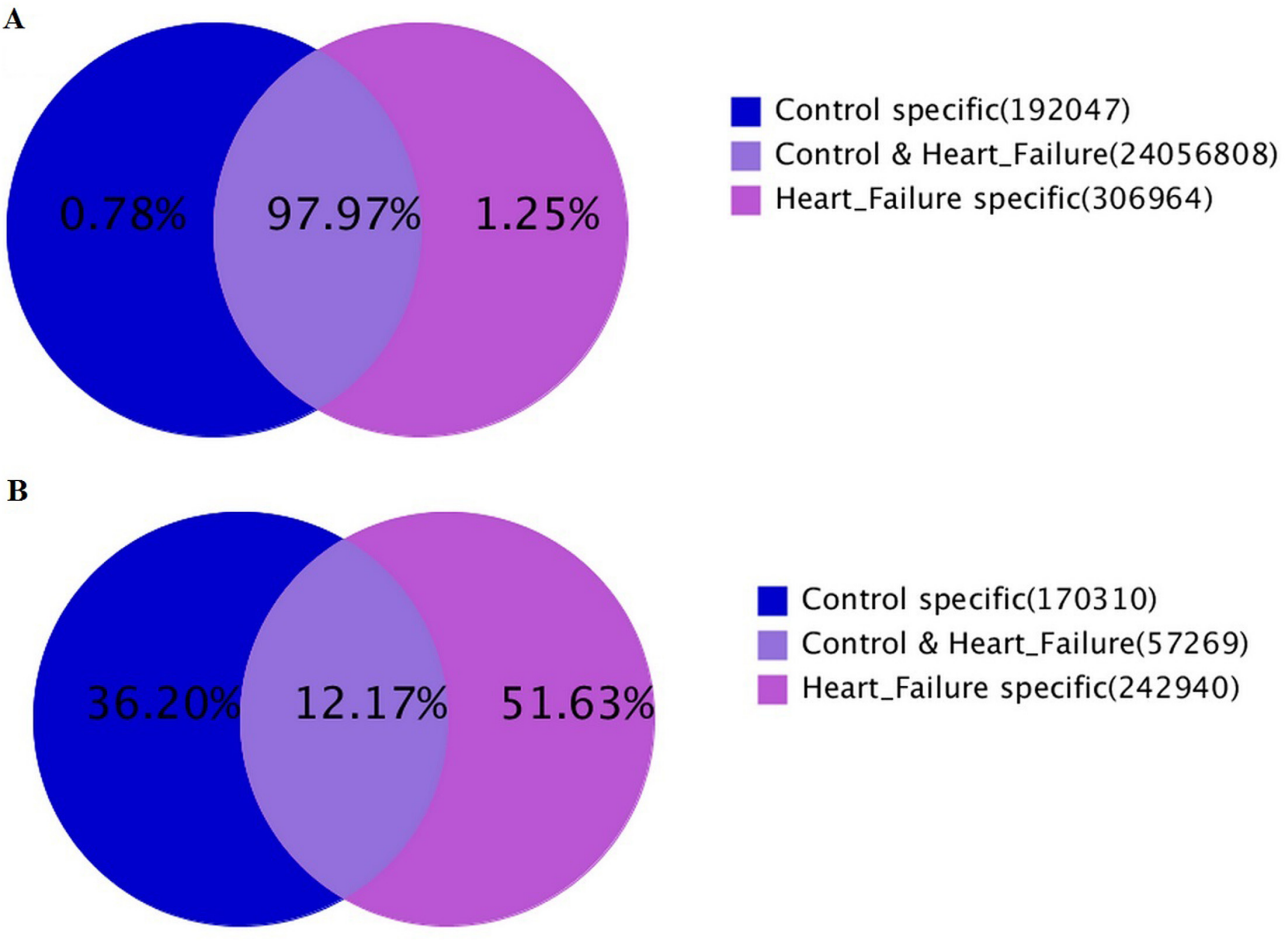
Summary of data cleaning and length distribution of tags. **(A)** Venn chart for total sRNA distribution. **(B)** Venn chart for unique sRNA distribution.

To obtain a comprehensive view of the expression of small RNAs in both HF (H) and control(C) rats’ hearts, solexa deep sequencing technology was employed on small RNA libraries from ventricle tissue. In all, 11927604 (C) and 12889151 (H) raw reads were generated from the two libraries respectively. After discarding the low-quality sequences, trimming the 3’ adapter and 5’ adapter sequences, 11822808 clean reads (99.12%, representing 227579 unique sequences) ranging from 14 to 29 nucleotides were generated for the C library, and 12733011 clean reads (99.07%, representing 300209 unique sequences) ranging from 12 to 31 nucleotides were generated for the H library. Notably, the number of unique sequences in the H library was over 1.3 fold more than that in the C library even though the total reads were approximately same in both libraries. As for the distributing of sRNAs in H and C library, 12.17% unique sRNAs fell into both libraries but they accounted for 97.97% of the total reads (Fig 2). The results indicated that the overall small RNA composition in the two groups was different, and the specific sRNAs, though expressed at low level,might play essential roles in the development of heart failure.

We further mapped these clean reads onto the rattus genome assembly with no mismatch using the SOAP program. After mapping, 67039 (C) and 106090 (H) unique sequences were represented by 6708481(C) and 7583745 (H) reads, respectively (Table 1). 2393 known miRNAs in the HF group and 2144 in the controls were identified, while 12 novel miRNAs in the HF and 26 novel ones in the control group were predicted. The most highly expressed miRNAs were rno-miR-1-3p, rno-let-7 family, rno-miR-29a-3p, rno-miR-133a-3p, rno-miR-499-5p and rno-miR-140-3p in both HF and control group.

**Table 1:** Small RNA sequence statistics for the control (C) and HF (H) libraries.

	# counts	% of total	# unique	% of total
H library				
Raw reads	12889151	-		
High-quality reads	12852634	100.00		
3′ adaptor sequence	5009	0.04		
5′ adaptor contaminants	42256	0.33		
Sequences < 18 nt removed	68921	0.54		
Poly A	80	0.00		
Clean reads	12733011	99.07	300209	
Clean reads	12733011	100	300209	100.00
Sequences mappingrattusdraft genome	7583745	59.56	106490	35.47
Known rattusmiRNAs	7351138	55.73	2393	0.80
Non-coding RNA (rRNAetc.)	224066	1.76	28880	9.6
Repeat-associated small RNAs	73891	0.58	9800	3.26
Unann	4981153	39.12	180317	60.06
C library				
Raw reads	11960005	-		
High-quality reads	11927604	100.00		
3′ adaptor sequence	4756	0.04		
5′ adaptor contaminants	70924	0.34		
Sequences < 18 nt removed	57878	0.49		
Poly A	41	0		
Clean reads	11822808	99.12	227579	
Clean reads	11822808	100.00	227579	100.00
Sequences mapping rattus draft genome	6708481	56.74	67039	29.46
Known rattusmiRNAs	6590964	55.75	2144	0.94
Non-coding RNA (rRNAetc.)	152299	1.29	22249	9.8
Repeat-associated small RNAs	35537	0.30	7406	3.3
Unann	4947214	41.84	141592	62.22

rRNAetc. Represents the total reads of four non-coding RNAs (rRNA, snRNA, tRNA and snoRNA).unann represents the remaining small RNAs which failed to be mapped by any of them.

**Fig 3:**
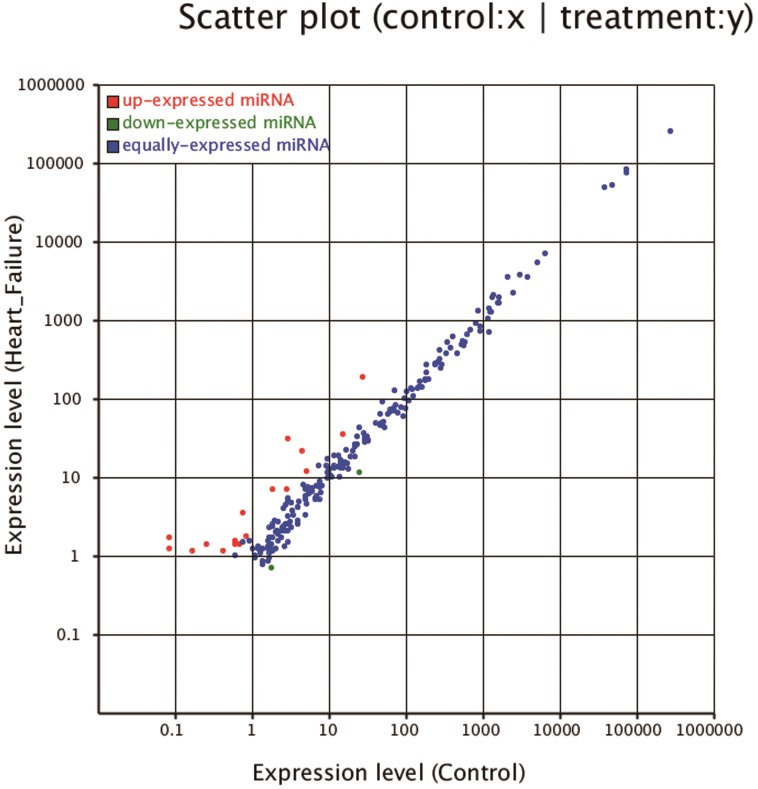
Scatter plot of the differential expression. Each plot represents a miRNA. Ratio: standard expression level(HF/C). Red plots: ratio>2; green plots: ratio<1/2; blue plots: 1/2≤ratio≤2.

The frequency of an individual miRNA can be used to compare the relative expression of miRNAs between libraries. Thus, we tested the differential expression of miRNAs in the HF and control libraries based on the normalized reads. In total, 106 known and/or homologous miRNAs showed differential expressions (p<0.05) between the HF and control samples; 78 miRNAs were significantly upregulated and 28 were downregulated in HF samples. Among them, 12 miRNAs (rno-miR-10b-5p, rno-miR-122-5p, rno-miR-184, rno-miR-1843-5p, rno-miR-196c-5p, rno-miR-199a-5p, rno-miR-202-5p, rno-miR-206-3p, rno-miR-208b-5p, rno-miR-224-5p, rno-miR-298-5p and rno-miR-31a-5p) were significantly upregulated(p<0.01, fold-change >1) compared to the control group and only rno-miR-208a-3p were significantly downregulated (p<0.01, fold-change <-1) (Fig 3). Among the top 13 differentially expressed miRNAs, the five most abundantly expressed miRNAs were rno-miR-122-5p, rno-miR-184, rno-miR-31a-5p, rno-miR-199a-5p and rno-miR-208a-3p. Meanwhile, rno-miR-10b-5p, rno-miR-184, rno-miR-1843-5p, rno-miR-196c-5p, rno-miR-202-5p, rno-miR-206-3p, rno-miR-224-5p, rno-miR-298-5p and rno-miR-31a-5p were reported for the first time to be differentially expressed in HF tissue.

### Verification of the miRNAs sequencing and detection of the dynamic expression pattern

**Fig 4:**
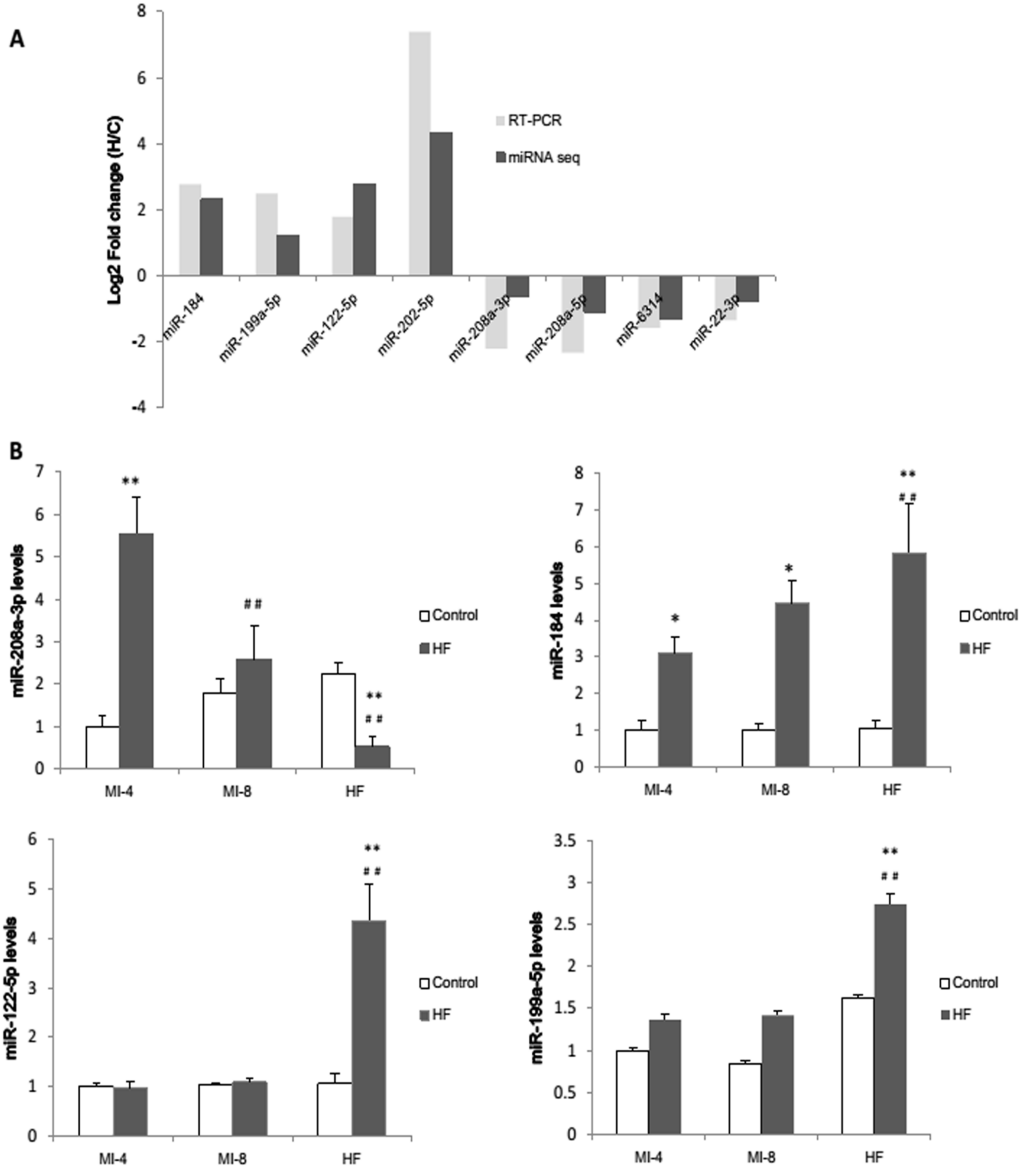
Prospective validation and time course analysis of miRNA sequencing analysis by qRT-PCR. **(A)**4 up-regulated miRNAs and 4 down-regulated miRNAs were selected for validation. The fold changes of selected miRNAs measured by qRT-PCR were statistically significant(p<0.05). miRNA expression difference was considered to be valid if the direction of change was the same. Time course analysis of miR-208a-3p **(B)**, miR-184 **(C)**, miR-122-5p **(D)** and miR-199a-5p **(E)** were studied. *p<0.05 and **p<0.01 vs the control group; ##p<0.01 vs the MI-4 group.

To confirm the results of the miRNA sequencing data, 4 upregulated miRNAs (rno-miR-122-5p, rno-miR-199a-5p, rno-miR-184 and rno-miR-202-5p) and 4 downregulated miRNAs (rno-miR-208a-3p, rno-miR-208a-5p, rno-miR-6314 and rno-miR-22-3p) were chosen to be further examined using real-time quantitative PCR. The miRNAs varied significantly in expression levels from less than 1 (rno-miR-631 in HF group) to more than 1200 (rno-miR-22-3p in control group) in the sequencing data, so thay could represent miRNAs of different expression levels. The fold change of the expression levels was calculated. The qRT-PCR results were consistent with our sequencing results (Fig 4A) which confirmed the accuracy of sequencing results.

To further confirm the results of the miRNA sequencing and analyze the dynamic expression pattern of specific miRNAs in HF rats, the dynamic changes of miR-208a-3p (Fig 4B), miR-184 (Fig 4C), miR-122-5p (Fig 4D) and miR-199a-5p (Fig 4E) in the process of post-infarcted heart failure were analyzed. The expression levels of the miRNAs in rat heart at 4 weeks, 8 weeks and 10 weeks post MI operation and the corresponding controls were examined respectively. The results showed that the expression of rno-miR-199a-5p and rno-miR-184 gradually increased with time after MI operation. The expression of rno-miR-122-5p was barely changed at the early stage of post-MI, but markedly increased when overt HF developed. There was a prominent increase of rno-miR-208a-3p in early stage of post-MI, but decreased gradually.

### Pathway analysis for the top 13differentially expressed miRNAs

The potential targets of the differentially expressed miRNAs were predicted using the miRGen database, which predicts targets using both PICTAR and TARGETSCANS. As a result, 71768 miRNA-mRNA interaction sites corresponding to 29115 target genes were identified which could be potentially regulated by the 13 top differentially expressed miRNAs. As expected, most of the miRNAs identified targeted hundreds of genes, of which 70% of the targets were regulated by more than one miRNA. Sik 2 (salt-inducible kinase 1) and Prkcb (protein kinase C, beta) were targeted by the highest number of miRNAs.

To fully understand the functions of the differentially expressed miRNAs, we performed a GO term and KEGG pathway annotation of the predicted miRNA targets using the DAVID gene annotation tool. Go term annotation results showed that protein phosphorylation, enzyme linked receptor protein signaling pathway, and regulation of signaling and regulation of cell communication were the most significantly enriched terms that has been known to regulate cellular physiological and pathological process. KEGG analysis identified 36 pathways that were over-represented, suggesting that these pathways are significantly regulated in the HF group. The pathways with the top 20 significant gene-enrichment are shown in table 2, which included “Wnt signaling pathway”, “Phosphatidylinositol signaling system”, “VEGF signaling pathway”, “Aldosterone-regulated sodium reabsorption”, “MAPK signaling pathway”, “Long-term depression” and “Gap junction”. Wnt/β-Catenin signaling has been reported to contribute to skeletal myopathy in heart failure through direct interaction with forkhead box O[24]. Phosphatidylinositol signaling system contributes to signal transduction, and VEGF signaling pathway and MAPK signaling pathway were highlighted to regulate heart fibrosis[25]. The pathways directly or indirectly regulate HF progression.

**Table 2:** Pathway analysis of the predicted targets of the 18 most differentially expressed miRNAs.

Pathway	Target genes with pathway annotation	Pvalue
Tight junction	552	1.66E-07
Wnt signaling pathway	362	5.00E-07
Pancreatic secretion	212	7.03E-07
Protein digestion and absorption	202	1.61E-06
Focal adhesion	566	6.19E-06
Adherens junction	224	9.54E-06
Bile secretion	124	1.18E-05
Phosphatidylinositol signaling system	256	1.61E-05
VEGF signaling pathway	195	1.76E-05
Melanogenesis	200	5.68E-05
Aldosterone-regulated sodium reabsorption	133	6.56E-05
Leukocyte transendothelial migration	351	7.59E-05
Meiosis–yeast	100	0.000106889
Oocyte meiosis	205	0.000154027
MAPK signaling pathway	558	0.000155636
Pathways in cancer	686	0.000224762
Long-term depression	157	0.000316915
Progesterone-mediated oocyte maturation	176	0.000320886
Cell cycle	194	0.000327893
Gap junction	171	0.00045674

### Effect of rno-miR-122-5p and rno-miR-184 on cell apoptosis

Based on the above-mentioned results, we found that rno-miR-122-5p and rno-miR-184 expression were increased during the development of heart failure. Apoptosis is one of the main causes of cardiac dysfunction in HF. In addition, miR-122-5p and miR-184 have been reported to be involved in the regulation of apoptosis under particular conditions, so we speculated that these two miRNAs could play important roles in the pathogenesis of heart failure through apoptosis.

**Fig 5:**
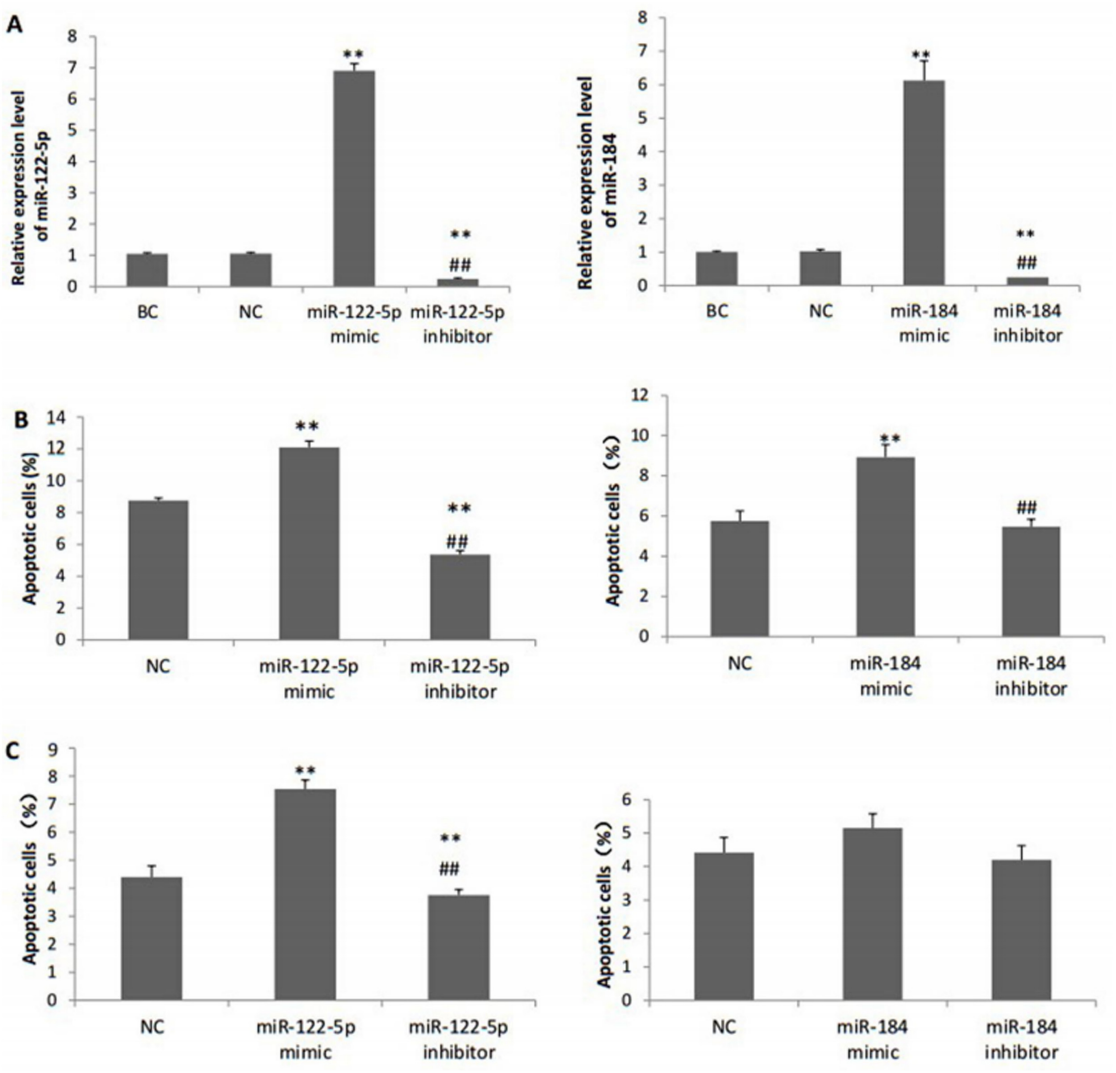
The apoptosis ratio detected by flow cytometry after transfection. **(A)** Relative expression levels of miR-122-5p and miR-184 after transfection. **(B)** Percentage of apoptotic cells after H_2_O_2_ treatment. **(C)** Percentage of apoptotic cells without H_2_O_2_ treatment. BC, blank control group; NC, negative control miRNA group.

We evaluated the H_2_O_2_-induced cardiomyocyte apoptosis using the MTS assay and flow cytometry. The gain-of–function and loss-of-function experiments indicated that miR-122-5p transfection could significantly promote cardiomyocyte apoptosis with H_2_O_2_ treatment (p<0.05) and even without H_2_O_2_ challenging (p<0.05). Transfection of miR-184 mimic could increase H_2_O_2_-induced cardiomyocyte apoptosis compared with the negative control (8.93% vs. 5.75%, p<0.05), and over-expression of miR-31a-5p in H9c2 cells without H_2_O_2_ treatment could only slightly increase apoptosis rate (6.54% vs. 5.43%, p>0.05). Transfection of miR-184 inhibitor had no obvious effect on apoptosis (p>0.05) (Fig 5).

## Discussion

The differential miRNA profiles of HF in a post-infarction HF rat model were determined, which could help to further define the potential miRNA regulatory networks during HF pathogenesis. In this study, 2939 known miRNAs and 7 novel miRNAs were identified with Solexa sequencing. We report here for the first time that 9 known miRNAs were significantly differentially regulated in HF group. In addition, we also analyzed the expression patterns of 4 miRNAs, explored the regulatory functions of miR-122-5p and miR-184during the process of HF pathogenesis.

Multiple evidences proved our profiling data reliable. First, different detection methods of miRNA expression levels have good consistency, such as high-throughput sequencing, microarray and qRT-PCR. Second, our results are largely concordant with previously reported findings. For example, rno-miR-1-3p, rno-let-7 family, rno-miR-29a-3p, rno-miR-133a-3p, rno-miR-499-5p and rno-miR-140-3p are most highly expressed in both HF and control group in our study, which was consistent with the previous studies that rno-miR-133, rno-miR-1 and rno-miR-499 are highly expressed in the heart[26], and miR-1, let-7 and miR-133 are highly expressed in the murine heart[27]. In addition, the overall abundance of the miRNAs in failing and non-failing myocardium differed not more than two-fold, which is in agreement with a recent study by Yang et al[28].

The miRNA signature of failing myocardium in our study indicated for the first time that 9 miRNAs were associated with HF, including rno-miR-184 and rno-miR-31a-5p, which were the most significantly differentially expressed between the two groups. Differential miR-184 expression has been reported to be responsible for the aberrant activation of Wnt signaling in ischemia-induced retinal neovascularization[29], which is consistent with our results that Wnt signaling pathway was significantly up-regulated in the HF group. It has been widely accepted that WNT signaling modulates mobilization of vasculogenic progenitors and differentiation of first heart field adult cardiac progenitor cells, thereby induces cardiac regeneration. On the other hand, WNT signaling upregulation could also aggravate left ventricular remodeling in HF, and WNT inhibition could attenuate left ventricular remodeling through inhibition of adult cardiomyocyte hypertrophy and apoptosis as well as LV fibrosis[30]. Wang et al[31] proved that oxidative modification of miR-184 participated in apoptosis through downregulating Bcl-xL and Bcl-w, which were not the native target of miR-184. However, the roles of non-oxidative miR-184 in apoptosis and heart failure have not been studied. Our results suggested that miR-184 expression was gradually increased during the post-infarction heart failure, which indicate that miR-184 regulated the pathogenesis of heart failure. In vitro results also found that miR-184 could promote apoptosis in H_2_O_2_ treated H9c2 cells. It was possible that miR-184 might play an important role in the regulatory networks of heart failure through apoptosis. Recent studies have found that miR-31 plays an important role in proliferation of vascular smooth muscle cells[32] and promotes the left ventricular remodeling of SHR. Katsure et al[33] reported that miR-31 played an positive role in endothelial-mesenchyme transition (EndMT) involved in development and pathogenesis through integrating TGF-β and TNF-α signaling. Our results suggested that miR-31a expression was increased in heart failure group, which indicated a potential role in the pathogenesis of heart failure.

MiR-208 family members are highly expressed in cardiomyocytes, and are closely related with ventricular remodeling [34]. The miR-208 family includes two subfamilies: miR-208a and miR-208b. It has been reported that miR-208a promotes heart failure progress through modulating cardiac fibrosis through increasing endoglin and collagen I expression[35, 36] and hypertrophy, and could be a biomarker for diagnosis and therapeutic target in treatment of heart failure[9, 37–39]. Although the pathways that directly related to fibrosis were not highlighted as the miRNA library was more likely to present myocytes instead of fibroblasts because of the high occupation, fibrosis was obviously significant in vivo. Whether or not miR-208 contributes to fibrosis is not fully elucidated. We found that in the heart failure group, miR-208b were up regulated, while the miR-208a-3p expression increased in the early stage after myocardial infarction, but decreased in the late stage, indicating that miR-208a-3p and miR-208b play different even opposite roles in heart failure, which needs to be clarified by further research.

Huang et at[40] found a potential role of miR-122-5p in cardiomyocyte apoptosis through Pax-8 knockout mice, which is consistent with our results that miR-122-5p expression increased in the late stage after infarction. Huang et al[40] proved that miR-122 contributes to apoptosis in H9c2 cells through participating the apoptotic gene expression. In our results, we found the apoptotic role of miR-122-5p in both normal and H_2_O_2_ treated H9c2 cells. We speculated that miR-122-5p participates in the heart failure progress via regulating apoptosis and could be a biomarker of heart failure. However, other studies found that the expression of miR-122 was downregulated in patients with severe fibrosis (SF) when compared with non-SF patients and controls, possibly through the upregulation of transforming growth factor beta 1 (TGF-b1)[41]. The opposite trends and effects of miR-122-5p in heart failure further demonstrated the complexity of miRNA regulation in diseases, and the exact function depends on different pathogenesis process and pathological state.

Van Rooij E et al[42] found that 7 miRNAs were upregulated in the heart failure sample, including miR-199a-5p,which promotes heart failure by regulating UPS[43]. These studies were consistent with our results of miR-199-5p in heart failure. Baμmgarten et al[44] found that TWIST1/miR-199/214 pathway is downregulated in the end-stage dilated cardiomyopathy, which might contribute to the loss of cardiac mass. In addition, Rane[45] suggested that knockdown of miR-199 induced proapoptotic genes upregulation, and replenishing miR-199a reduced apoptosis. However, we found miR-199 upregulated in the late post-infarction heart failure when more cardiomyocyte apoptosis occurred. These conflicting results probably reflect the diversified roles that miRNAs play in different pathological processes and states.

Among the predicted miRNA targets, *SIK 2* and *Prkcb* were assigned to the highest number of miRNAs. *SIK2* functions as a negative modulator of the insulin-dependent survival pathway and contributes to hyperglycemia-induced cell death of Muller glia. *Prkcb* plays a role in hexosamine biosynthesis pathway induced transcriptional regulation. Our analysis suggested that *Prkcb* is the target gene of 9 upregulated miRNAs, and is downregulated in the HF group, yet no related study has identified the effects and mechanisms of *Prkcb* on heart failure. *Prkcb* encodes protein kinase C β (PRKCB), and it has been proved that inhibition of PRKCB isoform ameliorates methylglyoxal advanced glycation end product-induced cardiomyocyte contractile dysfunction, possibly through modulation of oxidative stress, O2(-) generation, cell death, apoptosis and mitochondrial injury[46].We speculated that *Prkcb* could play a protective role in the HF progress.

The main limitation of the study was the small number of samples included. Second, the experiments to modulate miRNA expression in animals were not conducted to further prove the regulatory roles of related miRNA. Furthermore, the target genes and pathways of HF-associated miRNAs remain elusive and deserve further investigations.

## Conclusions

In conclusion, the current strudy indicated anetiological contribution of miRNAs in post-infarction heart failure and validated the potential use of miRNAs in future studies concentrating on the roles of individual miRNAs in the miRNA-based diagnosis and therapy. A more detailed understanding of the molecular mechanisms and regulatory pathways in heart failure are called for assistance in improving the diagnostic and therapeutic strategies.
